# Dorsal Dartos Flap Prepared Before Urethroplasty, Less Bleeding of Operation: A New Perspective on Hypospadias

**DOI:** 10.5152/tud.2022.22031

**Published:** 2022-03-01

**Authors:** Yasar Issi, Cemal Bilir

**Affiliations:** 1 Department of Urology, Bakırçay University Faculty of Medicine, İzmir, Turkey; 2 Department of Pediatric Surgery, Bakırçay University Faculty of Medicine, İzmir, Turkey

**Keywords:** Hipospadias, flap, bleeding time

## Abstract

**Objective:**

The benefits of preparing the dorsal dartos flap before urethroplasty were investigated.

**Materials and methods:**

Patients with coronal, subcoronal, and distal penile hypopadias without severe cordee who underwent surgical repair between October 2016 and September 2020 were included in the study. Tubularized incised plate urethroplasty technique was applied to all patients. The patients were divided into two groups: In Group 1, the dorsal dartos flap was prepared after urethroplasty, which is the commonly used technique today, and sutured on the neourethra. In Group 2, the dorsal dartos flap was prepared before the post-degloving urethroplasty. The amount of bleeding, the duration of the surgery, and the complications between the two groups were recorded and compared.

**Results:**

Twenty-two patients who could be followed up for at least 3 months were examined. There were 10 patients in Group 1 and 12 patients in Group 2. A statistically significant difference was found between the amount of bleeding of the operation in the two groups. Duration of operation, hematoma, infection, skin necrosis, or glanular dehiscence were not observed in any patient.

**Conclusion:**

Preparing the dorsal dartos flap, before urethroplasty significantly reduces the amount of bleeding. This may be a new modification alternative in hypospadias surgery.

**Main Points**

- In hypospadias surgery, preparing the dorsal dartos flap before urethroplasty can reduce the amount of bleeding of the operation.
- It can give this chance to surgeons who cannot apply the buttonhole technique to the flap due to their preferences during surgery.
- Its effect is not only on the amount of bleeding but also allows the dorsal dartos flap to be prepared comfortably when there is no bleeding from the glans.

## Introduction

Hypospadias is a disease in which there are many surgical techniques and personal preferences can change the surgical method.^1,2^ Tubularized incised plate urethroplasty (TIPU) and a protective layer using dartos flap became popular worldwide.^3,4^ There are still debates in the intermediate steps of this technique.^5-7^ The research and studies continue to improve the TIPU technique, reduce its complications, and get better penile appearance.

Dorsal dartos flap (DDF) is a widely used step with successful results today. It is prepared after urethroplasty, brought to the ventral surface with the buttonhole technique, and sutured as an additional layer on the neourethra. We hypothesized that the preparation of the DDF before urethroplasty would provide some benefits. The aim of this study was to prospectively investigate the effects of DDF preparation before and after urethroplasty on the amount of bleeding.

## Material and Methods

### Study Design and Inclusion Criteria

This is a single-blind prospective randomized controlled trial. Izmir Bakircay University ethics committee approved the protocol (approval ID: 339/319) and written informed consent was obtained from all the parents. The CONSORT guidelines were followed and a flowchart was performed for it. The number of individuals to be included in the study was made using the G*Power program. The sample size was calculated using posthoc power test, when study groups were adjusted as 15 patients we reached 40% power and *α *= 0.05 probability of error. Patients presenting with primary coronal, subcoronal, and distal penile hypospadia without severe chordee between October 2016 and September 2020 were included in the study. All operations were performed by a single pediatric urologist. None of the patients with chordee required additional interventions other than degloving. Patients who underwent penile plication, patients with secondary hypospadias, and patients with any additional comorbidities were excluded from the study. The amount of intraoperative bleeding, duration of operation, and complications in all the patients were recorded. The primary endpoint of the study was the occurrence of complications. The secondary endpoint was follow-up for 3 months postoperatively.

### Procedure

Preoperative antibiotic (intravenous cefazolin) prophylaxis was applied to all patients. Dorsal penile nerve block was performed with 0.5 mg/kg bupivacaine hydrochloride at the beginning of the operation. Chordee control was performed after degloving. All patients underwent the TIPU procedure. Dorsal dartos flap was applied as a top layer on the neourethra. The patients were divided into two groups: in Group 1, DDF was prepared after urethroplasty, which is the commonly used technique today, and sutured on the neourethra. In Group 2, DDF was prepared after degloving and before urethroplasty. A tourniquet was applied during urethroplasty in all repairs. As the beginning of the operation, complete degloving was determined and the end was determined as the last skin suture. In this way, the operative time of each case was recorded. The amount of intraoperative bleeding was measured with gauze pieces soaked in blood. The measurements were consistent with the literature, and it was determined that a 10 × 10 cm piece of gauze completely absorbed 5 mL of blood, and the number of gauze and the amount of bleeding were measured.^8^ All these measurements were made by a circulating nurse. A 6 fr balloon-free feeding tube was used for urethral catheterization in all patients. A self-adhesive elastic wrap was applied to the penis for 3 days.

### Randomization

The patients admitted for surgery were sequentially distributed to the groups. The patients were randomly assigned to the groups with flaps before urethroplasty and flaps after urethroplasty at a ratio of 1 : 1. The patients’ parents and the nurse who recorded the intraoperative study results were blinded to the study groups.

### Follow-up

The follow-up in the first, third, and sixth months were event-free in both groups. Meatus location, meatus stenosis, urethrocutaneous fistula, penile rotation, penile chordee, and other complications were examined.

### Statistical Analyses

Statistical analysis was performed using the Statistical Package for Social Sciences version 23 (IBM SPSS Corp.; Armonk, NY, USA). Normally distributed variables were compared using unpaired* t*-test, whereas the Mann–Whitney U-test was used for non-normally distributed variables. The normality of the distribution of the variables was evaluated with the Kolmogorov-Smirnov test. Chi-square and Fisher’s exact tests were used to test for the association between categorical variables. The probability value ≤.05 was considered to be statistically significant.

## Results

**Figure 1.**
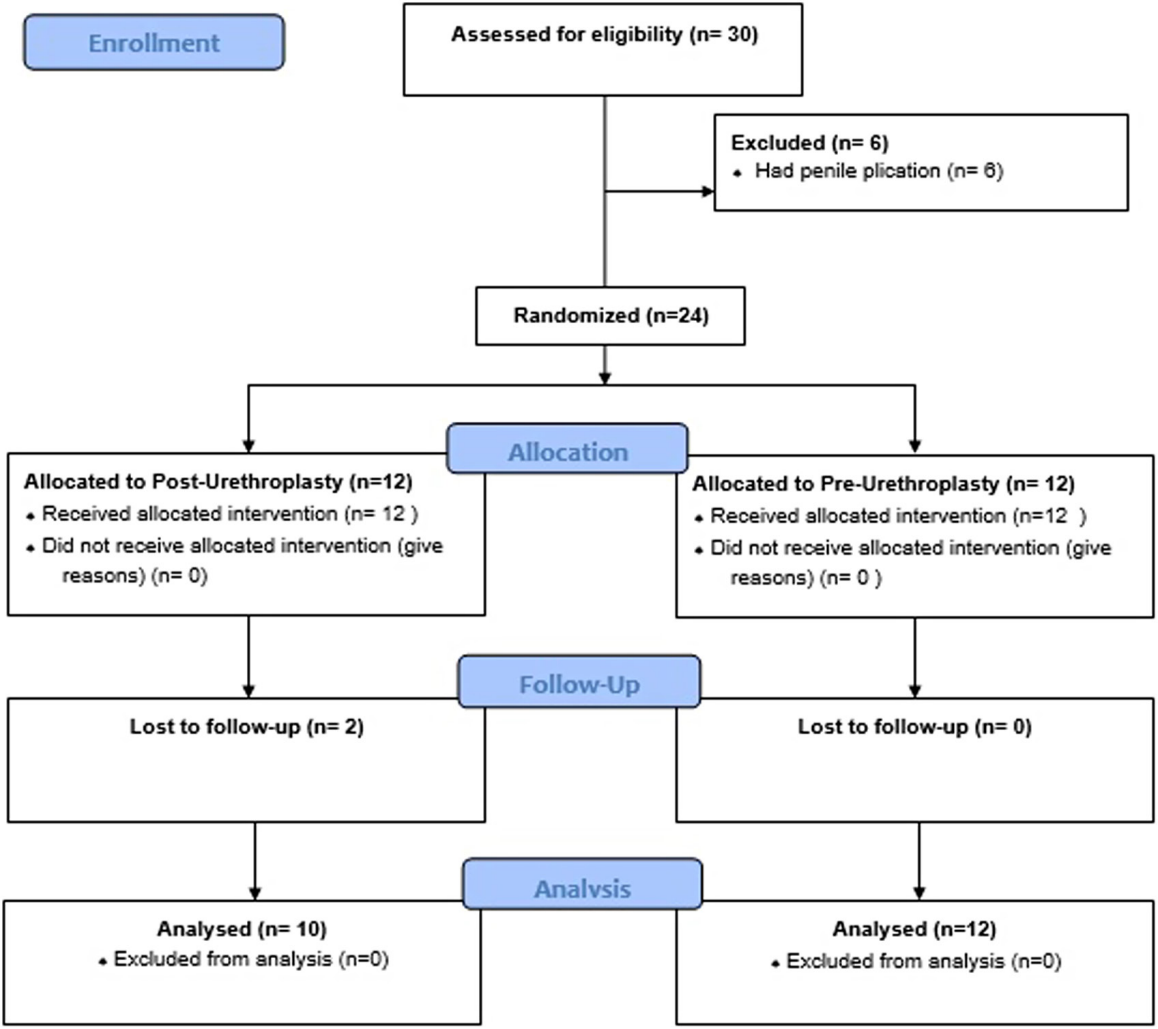
CONSORT flowchart of the study.

The CONSORT flow chart of the study is shown in Figure 1. Thirty patients were included in the study. Twenty-two patients who were followed up regularly for at least 3 months and did not meet the exclusion criteria were analyzed. There were 10 patients in Group 1 (standard technique, DDF prepared after urethroplasty) and 12 patients in Group 2 (DDF before urethroplasty). There was no significant difference between the groups in terms of age, meatus level, and complications. The amount of intraoperative bleeding in Group 1 (26.80 ± 15.78 mL) was significantly higher than the amount of bleeding in Group 2 (16.25 ± 3.76 mL) (*P = *.036). The number of bloody gauze pieces was also higher in Group 1 [median: 5 (min-max: 3-14) pieces—median: 3 (min-max: 2-5) pieces] (*P* < .001). While the duration of the operation was 61.80 ± 8.20 minutes in Group 1, it was 54.0 ± 3.79 minutes in Group 2 (*P* = 0.082) (Table 1). No hematoma, infection, skin necrosis, or glanular dehiscence was observed in any patient.

## Discussion

Hypospadias repair is a surgery in which all steps have not been fully standardized, except for certain points.^9^ The search continues to achieve less bleeding, fewer complications, and better surgical and cosmetic results. Since hypospadias surgery is mostly performed in young children, the amount of bleeding in this patient group is very important and can lead to various complications.^10,11^ Although some techniques (tourniquet and epinephrine application, use of some topical agents, dressing technique, etc.) are being studied to prevent this, there is no clear opinion as to which measure is most successful.

In a study by Alizadeh et al in which tourniquet application and epinephrine injection were compared, it was shown that epinephrine injection was more successful in reducing intraoperative bleeding.^10^ In a randomized controlled study, it was stated that the use of a tourniquet did not decrease the amount of bleeding, but it reduced the operative time and increased the satisfaction of young surgeons with regard to hemostasis.^12^ In an animal study, urothelial ultrastructural changes and cellular damage were investigated in rats that were not applied any hemostasis but applied an intermittent and continuous tourniquet for 30 minutes and received epinephrine injection. In the study, it was determined that these findings were more pronounced in the epinephrine group. No difference was found between intermittent and continuous tourniquet application.^13^ The epinephrine injection appears to be more effective, with some possible harmful effects.

In a randomized controlled study on the use and results of autologous platelet gels in hypospadias surgery, it was reported that bleeding was less in the gel group and there was no difference in the duration of surgery.^14^ In another study, it was shown that the topical application of feracrylum citrate was associated with significantly less bleeding compared to the control group.^15^

In an article investigating the surgical results of preoperative hormone use, the amount of bleeding was classified according to the need for an intraoperative tourniquet. Although tourniquet was used in more cases in the hormonal group, this could not be shown statistically.^16^ In a survey in which 99 pediatric surgeons and urologists participated, 56% of the physicians stated that preoperative hormone therapy caused an increase in intraoperative bleeding, while 44% stated that they did not see any difference.^17^

In a series of 356 cases by 2 surgeons, it was revealed that TIPU surgeries performed with ventral dartos flap took significantly shorter time than those performed with DDF, and complications of penile rotation and ventral skin necrosis were also higher in the DDF group.^6^

Bleeding during hypospadias repair is not only related to the “amount of bleeding” and the problems it may cause. In this surgery, in which microsurgical techniques are used, less bleeding ensures that the surgical area remains cleaner and provides suturation under clearer vision.

As all hypospadias surgeons know, the most intense bleeding begins when the glans is cut and continues during urethroplasty and dartos flap preparation. Because of of the fear of bleeding, some surgeons therefore do not perform the deeper glans dissection necessary. This leads to other major problems. Bipolar coagulation is useless at this stage. If the bleeding continues, it is usually tried to be brought under control with dressing techniques. If the bleeding is too much, it can lead to hematoma.^18^

At the present time, dartos flap is prepared after urethroplasty. In fact, the technique is correct as a step. However, the bleeding that started after the glans cutting continues because, the tourniquet was removed while preparing the DDF. Since the glansplasty is performed after the dartos flap, the glans remains open while the flap is being prepared and bleeding continues. In our modification, DDF is prepared after degloving. Thus, after urethroplasty, a ready flap can be sutured immediately onto the neourethra. Then, the surgery can be terminated with glansplasty and skin suturing. Therefore, less bleeding can be achieved.

Another advantage of this modification is for those who use a balloon catheter for hypospadias repairs. Surgeons who take the DDF to the ventral face with the buttonhole technique have a very difficult time passing the balloon catheter through this hole, so they either enlarge the hole and make the flap smaller or they divide the flap completely in the middle and bring it to the ventral face from both sides. Of course, dividing the flap into two can cause various problems: rotation problems, flap insufficiency, and so on. If DDF is prepared before urethroplasty as we have done, it is only a catheter-free penis that is passed through the buttonhole and it is quite easy.

Aside from statistical and technical differences, having a ready flap after urethroplasty, which takes a long time and requires attention, is a psychologically comforting situation for the surgeon. Preparing a necessary flap during the bleeding is a separate source of stress, therefore it is known that some surgeons do not prefer dartos flap.

There are some limitations of this study. First of all, the number of patients is relatively low. However, in a surgery such as hypospadias where there are many variables and directly affecting the outcome, exclusion criteria are kept very strict in order to keep most variables (chordee severity, level, number of surgeons, and technique applied) constant. Since the number of hypospadias patients who were operated during 4 years and also met the study criteria was low, we had to perform a posthoc power analysis. Apart from this, this modification is partially valid for surgeons using ventral flaps. Like the modification mentioned, they can still prepare the flap after degloving, but a ventral dartos flap can be prepared in the presence of a tourniquet after urethroplasty. However, this may cause long tourniquet time and in some cases, the poor quality or inadequate flap. Therefore, only patients who underwent DDF were selected in this study to avoid any confusion and statistical difference in terms of operative time.

In conclusion, preparing DDF before urethroplasty significantly reduces the amount of bleeding time. We recommend preparing the DDF before urethroplasty. Studies with more patients are needed to see more significant statistical differences on all variables.

## Author Contributions

Data Collection and/or Processing – Y.I., C.B; Analysis and/ or Interpretation C.B., Y.I.; Writing Manuscript: Y.I., C.B.

## Declaration of Interests

The authors have no conflicts of interest to declare.

## Figures and Tables

**Table 1.** Intraoperative and Postoperative Differences between the Two Groups

	Group 1 (n = 10) (After Urethroplasty)	Group 2 (n = 12) (Before Urethroplasty)	*P*
Age (year), median (min-max)	3 (1-11)	3 (1-7)	.709
Bloody gauze (number), median (min-max)	5 (3-14)	3 (2-5)	**.001**
Amount of bleeding (mL), mean ± std dev	26.80 ± 15.78	16.25 ± 3.76	**.036**
Duration of operation (min), mean ± std dev	61.80 ± 8.20	54.0 ± 3.79	.082
Follow up (month), mean ± std dev	9.4 ± 3.6	10.3 ± 3.3	.532
Fistula, n (%)	1 (10%)	1(8.3%)	
Meatus stenosis, n (%)	0	1 (8.3%)	
